# Cellular effects of outer membrane vesicles isolated from pks-positive strains of bacteria

**DOI:** 10.21203/rs.3.rs-9818453/v1

**Published:** 2026-06-11

**Authors:** Jeremy J. Colón-Morales, Yermary Morales-Lozada, Bismark Madera, Gabriela Báez-Bravo, Joel Wen Han Wong, Abel Baerga-Ortiz

**Affiliations:** University of Puerto Rico, Medical Sciences Campus; University of Puerto Rico at Río Piedras; University of Puerto Rico at Río Piedras; University of Puerto Rico at Río Piedras; Harvard University; University of Puerto Rico, Medical Sciences Campus

**Keywords:** colibactin, pks, megalocytosis, OMV, outer-membrane vesicles

## Abstract

The *pks island* is a gene cluster harbored by certain strains of gram-negative bacteria, that encodes the production of the genotoxic compound colibactin. Despite the importance of colibactin as an inducer of DNA damage, little is known about how it is transported into the affected cells. Here we explore the possible involvement of outer membrane vesicles (OMVs) in colibactin toxicity. We exposed HeLa cells to OMVs from NC101 (*pks*^+^) and from the NC101*ΔclbP* deletion mutant lacking the capacity to make mature colibactin. Some megalocytosis (~ 40% increase in nucleus size) was observed as a response to OMVs from NC101 after 72 hours. The OMVs from both wild-type NC101 and *ΔclbP* mutant were sufficient to cause a decrease in the number of viable cells after 4 and 72 hours. Additional effects include the activation of the DNA repair machinery. Images obtained through high-resolution confocal microscopy also reveal that labeled OMVs from all strains can enter the cell and locate inside the nucleus, causing a more pronounced enlargement vs. cells with no OMVs. In all, the damage caused by the isolated OMVs recapitulates some of the typical phenotypes associated with *pks*^+^ *E. coli*, although not all the effects were found to be *pks*-specific.

## Introduction

The *pks island* is a gene cluster present in some strains of commensal *E. coli* (specifically the B2 phylogroup) and in other proteobacteria^1,2^. The genes encoded within this cluster drive the synthesis of a polyketide/non-ribosomal peptide (PK/NRP) compound colibactin, which has been implicated in tissue damage and in the development of colorectal cancer^3,4^. Colibactin is the final product of a series of *Claisen*-like condensation reactions that take place in the bacterial cytosol where it is made as an inactive precursor termed pre-colibactin^5,6^. Pre-colibactin is then activated by the peptidase ClbP by a hydrolytic de-acylation which provokes an intramolecular re-arrangement that results in its activation^7^. It has been shown that the deletion of the *clbP* gene results in the abolishment or attenuation of phenotypes typically associated with the toxin, highlighting the key role played by this peptidase enzyme in the maturation and activity of colibactin^8–10^.

There are numerous cellular phenotypes associated with colibactin toxicity and the most evident one is megalocytosis^1^. The megalocytic phenotype was first described in the liver cells of bovine cattle following ingestion of toxic compounds present in the feed^11,12^. Megalocytic cells are easily identified by their enlarged nuclei that can be readily seen in a microscope^1,13^. Strains of *E. coli* harboring the *pks* genes, are capable of such damage, which is typically followed by cell cycle arrest (specifically G2/M transition). The cells, in turn, become senescent as a result of the DNA damage via double strand-breaks (DSBs) caused by attempted repair through the Fanconi anemia pathway resulting in interstrand crosslinks^1,14,15^, thus activating the typical markers of DNA damage: γH2AX, CHK1, and CHK2^1,16^. Thus, contact with *pks*^+^ bacteria can cause extensive damage to the cellular genome and to its ability to control division.

While most of the work to elucidate the mechanism of action of colibactin has centered on the structure and reactivity of the molecule, there are unanswered questions on how colibactin is transported from the bacteria to the mammalian cell. Among the known routes of delivery for bacterial toxins, one that has generated much interest is outer membrane vesicles (OMVs)^16,17^. OMVs are sphere-like nanoscale particles released by gram-negative bacteria, that are involved in the transport of outer membrane and periplasmic content^23,24,25^. OMVs have several described functions, such as the delivery of proteins, virulence factors, bacterial survival factors, horizontal gene transfer, and immunomodulation^20–23^. Some of the cargo transported in these vesicles can either be functional substances with a specific target or waste material such as misfolded proteins^24^. Therefore, OMVs are an essential, dynamic, and multifunctional tool for bacterial survival and competition.

OMVs from certain *E. coli* strains, even from non-pathogenic bacteria, can cause DNA damage and promote diverse responses in mammalian cells upon contact^25,26^. Interestingly, these outcomes are similar to those attributed to *pks island* and colibactin. Thus, an attractive hypothesis is that OMVs could play a role in the delivery of colibactin to mammalian cells^22^.

In this work, we report the effects of bacterial OMVs on mammalian cells, focusing on the phenotypes typically attributed to colibactin exposure. We exposed HeLa cells to OMVs isolated from colibactin-producing bacteria, clinical isolate *E. coli* NC101, and from its corresponding *clbP* deletion mutant, which cannot produce mature colibactin, to measure the effects on nuclear size and the relative expression of common DNA damage biomarkers. Additionally, we labeled OMVs with fluorescent tags to trace them by confocal microscopy. We found that OMVs by themselves can increase cell and nucleus size. We also found that OMVs from *pks*^+^ *E.coli* were more highly correlated with the activation of DNA damage repair systems and with a reduction in the number of cells in culture. In summary, some but not all, of the effects associated with colibactin exposure were recapitulated by exposure to OMVs.

## Results

### Physicochemical Characterization of OMVs by Dynamic Light Scattering (DLS) and Confocal Microscopy.

**Figure 1:**
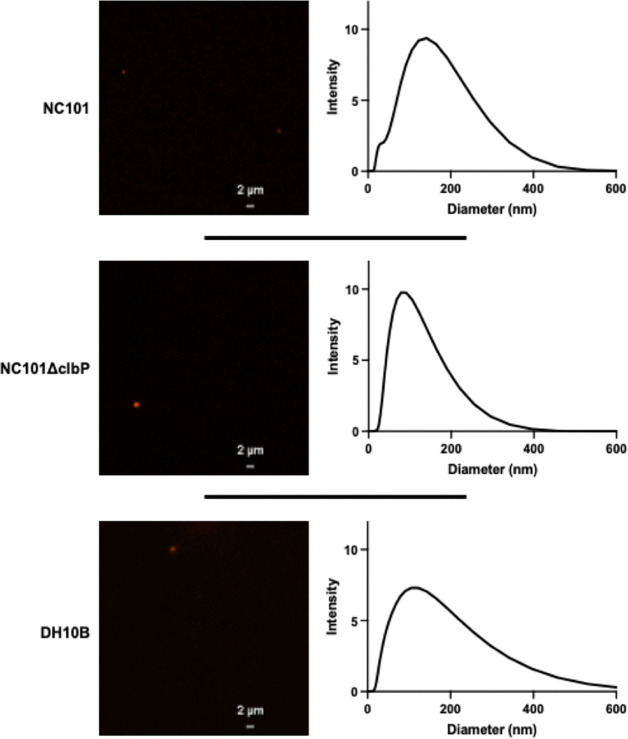
Confocal imaging of Vybrant^®^ DiI-labelled OMVs and their size distribution graphs through DLS. Images on the left panels were captured on a confocal microscope and show fluorescently labeled OMVs in suspension (red dots) while the graphs on the right panels present the size distribution of each of the OMV samples obtained by dynamic light scattering.

OMVs were isolated from liquid cultures of *E. coli* and quantified by measuring protein content in samples through a BCA Assay. The average concentration of OMVs were 83.0 ± 3.0 μg/mL for NC101 OMV samples, 76.2 ± 0.3 μg/mL for NC101Δ*clbP* OMV samples, and 99.6 ± 2.6 μg/mL for DH10B OMV samples (Table 1). The presence of OMVs in the cell-free supernatants of NC101, NC101Δ*clbP*, and DH10B *E. coli* strains was confirmed by DLS and confocal microscopy (Fig. 1). The average diameters of OMV samples were 135.3 ± 9.7 nm for NC101, 164.3 ± 31.4 nm for NC101Δ*clbP*, and 122.4 ± 0.0 nm for DH10B (Table 1).

### OMVs from E. coli NC101 cause megalocytosis in a human cell line.

**Figure 2:**
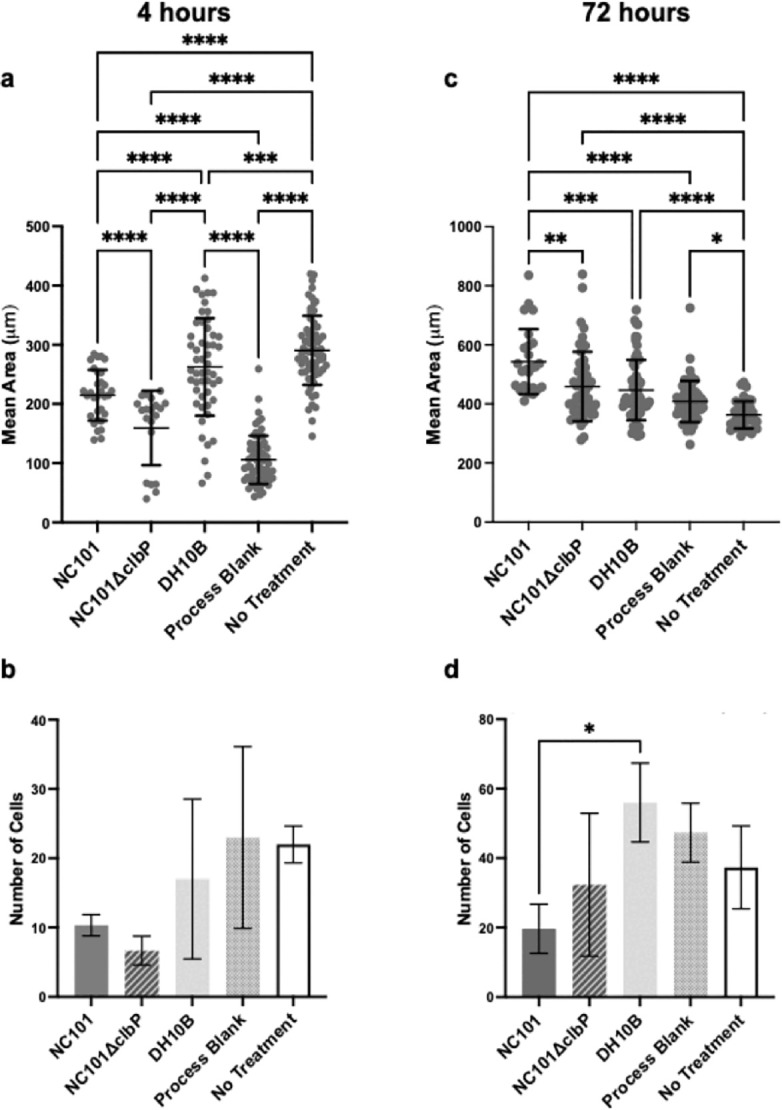
Effects of bacterial OMVs on HeLa cell nuclear area and cell count. **a.** Average nuclei area measurements after a 4-hour exposure. The average nuclei area were 214.8 ± 42.8 μm^2^, 159.4 ± 62.8 μm^2^, 262.6 ± 82.4 μm^2^, 105.8 ± 40.6 μm^2^, and 290.6 ± 58.4 μm^2^ for NC101, NC101Δ*clbP*, DH10B, Process Blank, and No Treatment, respectively. **b.** Average HeLa cell count between the 3 wells for each OMV treatment in the 24-well plate after a 4-hour exposure. Average HeLa cell counts were 10 ± 2, 7 ± 2, 17 ± 12, 23 ± 13, and 22 ± 3 for NC101, NC101Δ*clbP*, DH10B, Process Blank, and No Treatment, respectively. **c.** Average nuclei area measurements of HeLa cells exposed to bacterial OMVs for 4 hours and then allowed to recover for 68 hours. The average area of the nuclei measured were 551.4 ± 156.1 μm^2^, 454.9 ± 151.1 μm^2^, 446.6 ± 156.8 μm^2^, 412.7 ± 109.3 μm^2^, and 365.5 ± 83.0 μm^2^ for NC101, NC101Δ*clbP*, DH10B, Process Blank, and No Treatment, respectively. **d.** Average HeLa cell count between the 3 wells for each OMV treatment in the 24-well plate after a 4-hour exposure and 68-hour recovery period. Average cell counts were 20 ± 7, 32 ± 21, 56 ± 11, 47 ± 9, and 37 ± 12 for NC101, NC101Δ*clbP*, DH10B, Process Blank, and No Treatment, respectively. Confidence level was α = 0.05 and p-values were: * > 0.0332, ** > 0.0021, *** > 0.0002, **** < 0.0001.

The capacity of OMVs from all *E. coli* strain to induce megalocytosis of HeLa cells was measured in triplicates. Results show that after a 4-hour exposure to bacterial OMVs there is no discernible effect on nucleus size (Fig. 2a). Although the p-values for these measurements were within the acceptable range, indicating some statistical significance, none of the treated cells showed nuclear enlargement vs. cells that received no treatment. Exposure to OMVs seems to have affected the number of cells present after a 4-hour OMV exposure, with fewer cells counted in the presence of OMVs from NC101 or NC101Δ*clbP* (Fig. 2b).

Following a 4-hour exposure and a subsequent 68-hour recovery time, there were significant differences in nucleus size in cells exposed to NC101 OMVs when compared with the nuclei of cells exposed to NC101Δ*clbP* OMVs, DH10B OMVs, the Process Blank, and untreated cells (p = 0.0015, p = 0.0004, p < 0.0001, and p < 0.0001, respectively) (Fig. 2c). The cell counts reveal a larger effect on growth for cells exposed to OMV from NC101 and NC101Δ*clbP* strains, with a statistically significant difference observed in the amount of cells present in wells treated with NC101 OMVs vs. cells treated with DH10B OMVs (p = 0.0371). (Fig. 2d).

### OMVs from E. coli translocate into the cell nucleus.

**Figure 3:**
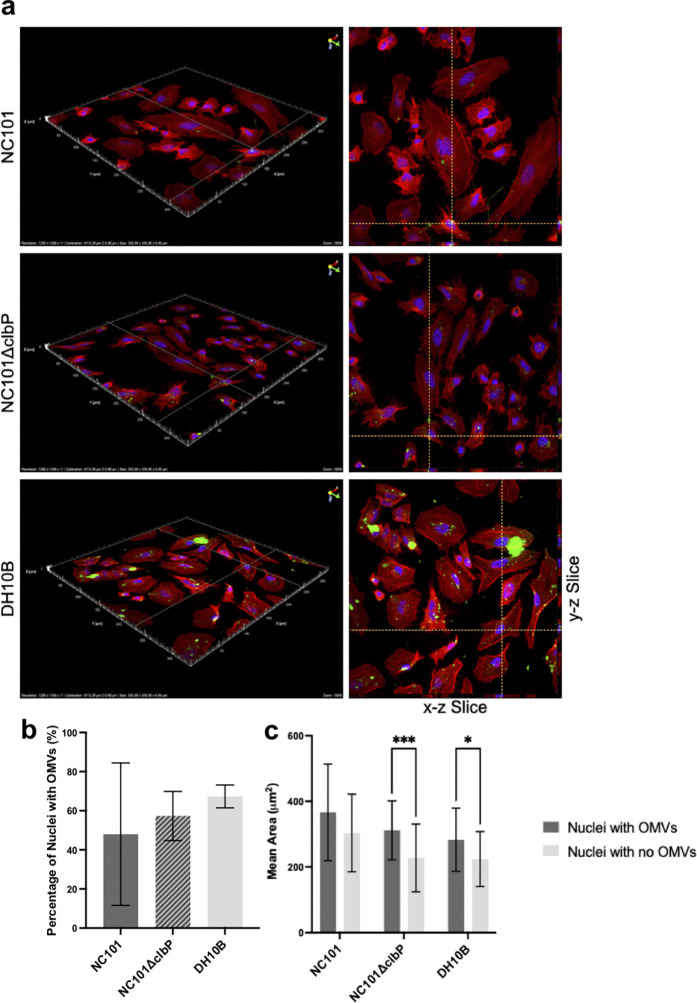
Bacteria OMV localization in HeLa cells after a 4-hour exposure. **a.** Three-dimensional images of cells exposed to OMVs from *E. coli* strains NC101, NC101Δ*clbP*, and DH10B (Left). Images were cross-sectioned across the x-z plane (bottom slice) and the y plane (right slice) (Right). OMVs in the nuclei are highlighted in slices from each image (yellow dotted cross). **b.** Percent of cells that have OMV present in the nucleus vs. the total of cells with internalized OMVs following a 4-hour exposure. The total number of cells that internalized NC101, NC101Δ*clbP*, or DH10B OMVs were 47, 70, and 61, respectively. The percent of cells exhibiting OMV fluorescence in their respective nuclei were 48.0% ± 36.4%, 57.3% ± 12.6%, and 67.3% ± 5.9%, for cells treated with OMVs from strains NC101, NC101Δ*clbP*, or DH10B, following a 4-hour exposure. **c.** Average area of nuclei from HeLa cells that contain OMVs compared with those that did not contain visible OMVs after a 4-hour exposure. Mean area for nuclei with OMVs were 366.4 ± 147.3 μm^2^, 311.6 ± 89.9 μm^2^, and 282.9 ± 96.1 μm^2^ for NC101, NC101Δ*clbP*, and DH10B, respectively. Mean area for nuclei with no OMV internalization were 303.03 ± 118.5 μm^2^, 227.3 ± 103.2 μm^2^, and 224.0 ± 83.7 μm^2^ for NC101, NC101Δ*clbP*, and DH10B, respectively. Confidence level was α = 0.05 and p-values were: * > 0.0332, ** > 0.0021, *** > 0.0002, **** < 0.0001.

The internalization of OMVs into cells and further localization into the cell nucleus was assessed via fluorescent confocal microscopy. Cross-sections of confocal images were done to assess the localization of the DiO-labelled OMVs in cells (Fig. 3a). The number of nuclei that contained OMV-specific fluorescence was counted and analyzed in relation to the total amount of cells with internalized OMVs in the cytosol. Following a 4-hour exposure, 47 cells exposed to NC101 OMVs presented OMV internalization, 48.0% of which presented OMV fluorescence inside the nucleus; 70 cells exposed to NC101Δ*clbP* OMVs presented OMV internalization, 57.3% of which contained OMVs inside the nucleus; and 61 cells exposed to DH10B OMVs presented OMV internalization, 67.3% of which contained OMVs inside the nucleus (Fig. 3b). The areas of the nuclei that contained OMV-specific fluorescence after the 4-hour exposure were measured and compared with the area of nuclei that did not contain OMV-specific fluorescence (Figs. 3c). Some statistical significance could be observed in mean area of HeLa cell nuclei that had internalized OMVs from NC101Δ*clbP* (p = 0.0003) and from DH10B (p = 0.0230).

**Figure 4:**
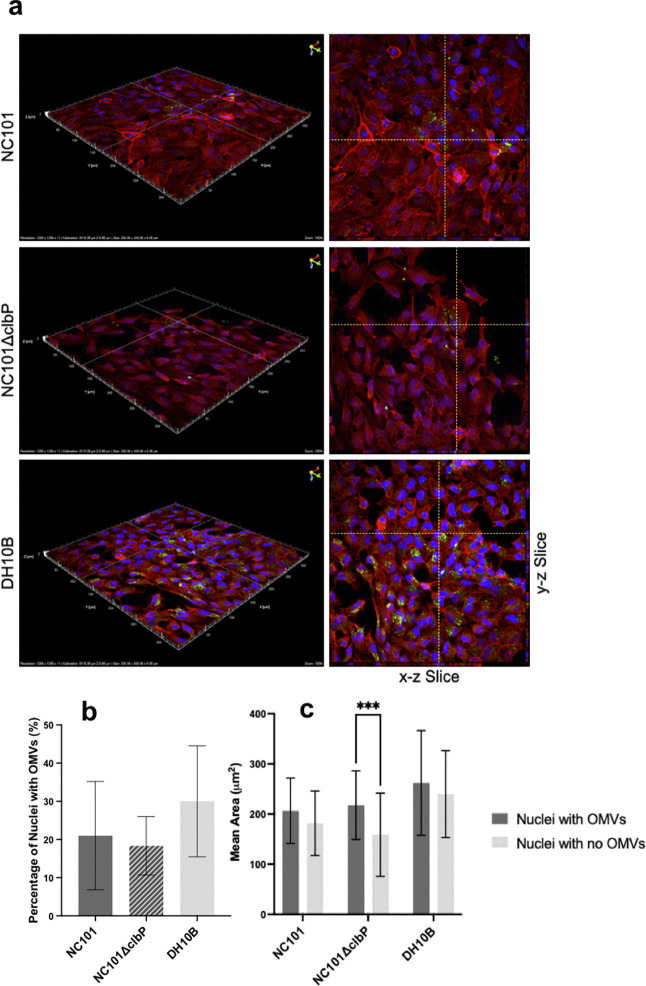
Bacteria OMV localization in HeLa cells after a 4-hour exposure and 68 hours of recovery to bacterial OMVs. **a.** Three-dimensional images of cells exposed to OMVs from *E. coli* strains NC101, NC101Δ*clbP*, and DH10B (Left). Images were cross-sectioned across the x-z plane (bottom slice) and the y-z plane (right slice) (Right). OMVs contained within nuclei are highlighted in slices from each image (yellow dotted cross). **b.** Percentage of cells that have nuclei containing OMVs to cells that internalized OMVs only into their cytosol OMVs following a 4-hour exposure and a 68-hour recovery period. The total number of cells that internalized NC101, NC101ΔclbP, or DH10B were 144, 119, and 194, respectively. The percentages of nuclei from those cells containing OMVs from strains NC101, NC101Δ*clbP*, or DH10B, following a 4-hour exposure were 21.0% ± 14.2%, 18.3% ± 7.6%, and 30.0% ± 14.5%, respectively. **c.** Average area of nuclei from HeLa cells that have internalized OMVs compared with those that had not internalized OMVs. Mean area for nuclei with OMV internalization were 206.4 ± 65.2 μm^2^, 217.5 ± 68.4 μm^2^, and 262.0 ± 104.2 μm^2^ for NC101, NC101Δ*clbP*, and DH10B, respectively. Mean area for nuclei with no OMV internalization were 181.6 ± 64.3 μm^2^, 158.8 ± 82.9 μm^2^, and 239.8 ± 86.6 μm^2^ for NC101, NC101Δ*clbP*, and DH10B, respectively. Confidence level was α = 0.05 and p-values were: * > 0.0332, ** > 0.0021, *** > 0.0002, **** < 0.0001.

Following a 4-hour exposure and a subsequent 68-hour recovery period, cross section images were taken to track OMV localization in cells (Fig. 4a). A total of 144 cells exposed to NC101 OMVs presented internalization, 21.0% of which contained OMVs inside the nucleus; 119 cells exposed to NC101Δ*clbP* OMVs presented OMV internalization, 18.3% of which contained OMVs inside the nucleus; and 194 cells exposed to DH10B OMVs presented OMV internalization, 30.0% of which contained OMVs inside the nucleus (Fig. 4b). The area of nuclei from HeLa cells that contained OMVs after a 4-hour exposure and a 68-hour recovery period, were measured and compared with the area of nuclei that did not contain OMVs (Fig. 4c). Significant differences could be observed in mean nuclei area of HeLa cells that contained OMVs from NC101Δ*clbP* (p = 0.0004).

### OMVs from E. coli NC101 induce DNA damage and double strand breaks.

**Figure 5:**
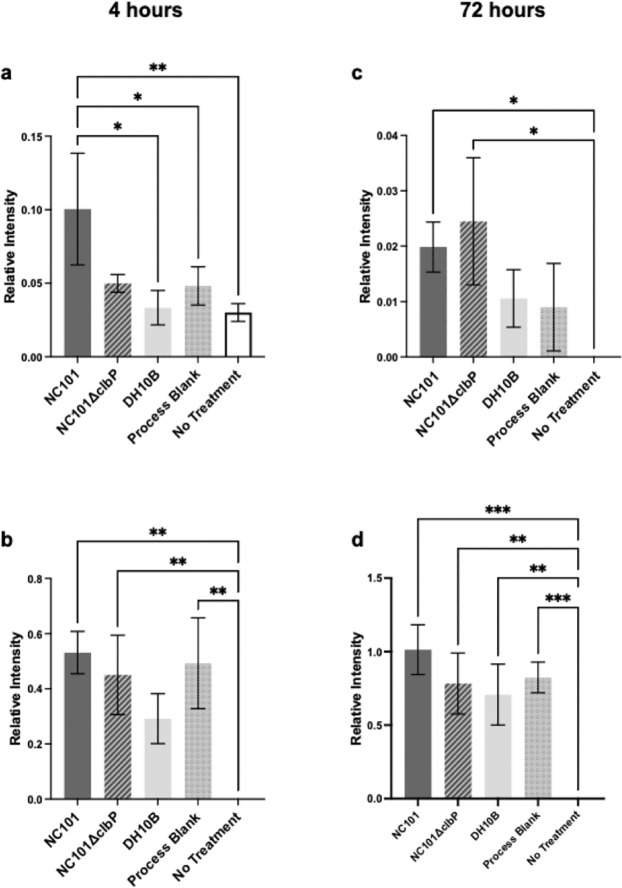
Relative expression of DNA damage biomarkers in HeLa cells exposed to bacterial OMVs. **a.** Relative expression of p-Chk2 after a 4-hour exposure. **b.**Relative expression of p-H2AX after a 4-hour exposure. **c.** Relative expression of p-Chk2 after a 4-hour exposure and a 68-hour recovery period. **d.**Relative expression of p-H2AX after a 4-hour exposure and a 68-hour recovery period. Confidence level was α = 0.05 and p-values were: * > 0.0332, ** > 0.0021, *** > 0.0002, **** < 0.0001.

The capacity of OMVs isolated from different *E. coli* strains to induce DNA damage was assayed by quantitative western blots of markers associated with DNA damage: histone γ-H_2_AX, Chk1, and Chk2. The increased phosphorylation of the histone γ-H_2_AX serves as a marker of DNA double-stranded breaks specifically, whereas Chk1 and Chk2 phosphorylation are general markers of DNA damage. Average relative expression of p-Chk2 following a 4-hour exposure to bacterial OMVs was 0.100 ± 0.038, 0.050 ± 0.006, 0.033 ± 0.012, 0.048 ± 0.013, and 0.030 ± 0.006 for NC101-, NC101Δ*clbP*-, DH10B, Process Blank, and untreated cells, respectively (Fig. 5a). For p-H2AX, relative expression was 0.531 ± 0.077, 0.450 ± 0.144, 0.292 ± 0.091, 0.493 ± 0.164, and 0 for NC101-, NC101Δ*clbP*-, DH10B-, Process Blank-, and non-treated cells, respectively (Fig. 5b). The expression of p-Chk1 could be detected in any of the cell samples.

The expression of DNA damage markers was also measured after a 4-hour exposure to bacterial OMVs and a subsequent recovery period of 68 hours. The relative intensities for the expression of p-Chk2 a was 0.020 ± 0.005, and 0.024 ± 0.011, 0.011 ± 0.005, 0.009 ± 0.008, and 0 for NC101-, NC101Δ*clbP*, DH10B, Process Blank, and untreated cells, respectively (Fig. 5c). For p-H2AX, relative expression was 1.013 ± 0.169, 0.783 ± 0.207, 0.707 ± 0.207, 0.825 ± 0.104, and 0 for for NC101-, NC101Δ*clbP*-, DH10B-, Process Blank-, and non-treated cells, respectively (Fig. 5d). The expression of p-Chk1 could be detected in any of the cell samples.

## Discussion

The *pks* genomic island encodes the production of colibactin which causes double strand breaks and interstrand crosslinks in the DNA of mammalian cells, which leads to megalocytosis and cell cycle arrest. The presence of *pks*^+^ *E. coli* has been linked with bacteria-induced colorectal cancer in humans^3,4,27–30^. Despite all the knowledge that have been gained on the biosynthesis and mode of action of colibactin, there are still questions about how colibactin is transported from the bacterial periplasm to the target cell, especially since the active form of colibactin has never been recovered from *E. coli* supernatants. This work explores the role of bacterial membranes in mediating the deleterious effects of colibactin, especially since OMVs by themselves have been shown to play a variety of roles^19^, including the delivery of virulence factors and toxins.^31,32^ Our starting hypothesis was that OMVs are a likely mediator of colibactin toxicity. In this work, we report that OMVs from a *pks*^+^ *E. coli* strain NC101 are sufficient for the elicitation of some of the hallmarks of the colibactin phenotype, including megalocytosis and the up-regulation of the DNA repair markers, particularly those involving double strand breaks. However, we also observed effects such as the localization of OMVs in the cell nucleus, which were not strain-specific.

We performed a detailed analysis on the size distribution of our isolated OMVs. The OMVs from the NC101 strains were on average larger in size than those isolated from the non-pathogenic strain according to results from Dynamic Light Scattering (DLS) (Table 1), which revealed the presence of particles with diameters (~ 125 to ~ 195 nm interval diameter) that fall within the expected range of sizes^19,33,34^. Additionally, we confirmed the presence of OMVs through confocal microscopy imaging of fluorescently-labeled OMVs using a lipophilic tracer molecule (Fig. 1). The size of OMVs has shown to be important for pathogenicity. A recent study revealed that larger OMVs from pathogenic bacteria cause a higher degree of double strands breaks and DNA damage on colonic epithelial cells^31^. Also, in cancer-causing pathogen *H. pylori*, it has been shown that vesicle size plays a role in how OMVs enter epithelial cells and cause damage^35^. Our results show that OMVs extracted from both clinical strains NC101 and NC101Δ*clbP*, are larger in size that those extracted from the non-pathogenic DH10B, an observation that is consistent with the size-dependent activity of OMVs. However, these size differences were not associated with the production of active colibactin, since the Δ*clbP* mutant produced the largest OMVs. In all, these data could suggest that the deletion of *clbP* could cause the secretion of larger OMVs from bacteria possibly due to the accumulation of pre-colibactin species in the periplasmic space.

The deletion of *clbP* has been reported and validated both in the probiotic strain Nissle 1917 and in DH10B harboring a bacterial artificial chromosome (BAC) that contains all the *pks* genes except for *clbP*. In both of these previous instances, the deletion of *clbP* resulted in a complete elimination of the colibactin phenotype. In this work, we compared the genotoxicity of a wild-type strain NC101 with that of a deletion mutant incapable of producing colibactin, NC101Δ*clbP*. To our surprise, the deletion of *clbP* did not completely abolish the megalocytosis phenotype as expected from the reported results of live *E. coli*.^10,36^ Despite this lack of a clear effect, the cells responded slightly more to OMVs from the wild type NC101 that to OMVs extracted from the NC101Δ*clbP* mutant.

Prior studies had shown that OMVs from many different strains of *E. coli*, including some *pks*^+^ strains, are internalized via clathrin-dependent endocytosis^37^. However, it had not been shown that *pks*+ OMVs can localize inside the nuclear envelope^38^. In this work, we used high-resolution confocal microscopy to follow OMVs as they entered the exposed HeLa cells. Our confocal images reveals that OMVs are mostly localized in the perinuclear region of treated cells and that some of them penetrate the nuclear envelope (Figs. 3 and 4), as has been reported for other pathogenic bacteria^25,39^. This level of entry into the nucleus seems to correlate with the degree of nuclear enlargement observed (Fig. 3c and 4c).

In conclusion, this work shows that OMVs from a colibactin-producing *E. coli* are sufficient to induce the DNA damage response in HeLa cells. Moreover, it was determined that OMVs from a mutant strain of the E. coli NC101 deficient of *clbP* gene had decreased toxicity, but still elicited some of the phenotypes attributed to colibactin-producing bacteria. Further work will be required to fully ascertain the involvement of OMVs as mediators of colibactin genotoxicity.

## Experimental procedures

### Bacterial strains.

*E. coli* NC101 *pks*^+^, and NC101Δ*clbP* were originally made and reported in Arthur et al., 2012^3^. *E. coli* DH10B *pks*^−^ were purchased from ThermoScientific.

### Cell culture.

HeLa cells (ATCC CCL-2) were cultured in Dulbecco’s Modified Eagle’s Medium (DMEM; SIGMA) supplemented with 10% fetal bovine serum (FBS) (SIGMA) and Penicillin/Streptomycin (100 U:100 μg/mL) (CORNING). Culture was done at 37 °C with 5% CO_2_. Cells were grown in T25 flasks until the fourth passage. HeLa cells were counted in a BioRad TC20^™^ Automated Cell Counter using Trypan Blue. A total of 1.0 × 10^4^ cells were transferred to individual wells in a 24-well plate.

### Isolation of OMVs.

OMV isolation was performed following a modified version of the Qiagen exoEasy Maxi Kit protocol for exosome isolation.^40^ Briefly, 100 mL bacterial cultures were divided into five 20 mL samples of bacterial culture that were centrifuged at 3,000 g for 30 minutes to allow bacterial pellet formation. The bacterial culture supernatant was filtered through Millipore^®^ Millex^™^ PVDF syringe filter, 0.45 μm^2^ pore size to remove residual bacteria that may not have adhered to the pellet. Filtered supernatant was then mixed with Qiagen Buffer XBP at a 1:1 ratio and carefully mixed by inversion 5 times. The filtered supernatant:Buffer XBP mixture was placed in exoEasy Maxi columns and centrifuged at 500 g for 1 minute. The flowthrough was discarded, and the column was washed once with Qiagen Buffer XWP by adding 10 mL to the column and centrifuging at 3,000 g for 5 minutes. For OMV elution from the column, 500 μL of Qiagen Buffer XE was added onto the column and allowed to incubate for 5 minutes. The column was centrifuged at 500 g for 5 minutes to elute OMVs. This process was repeated once more to obtain a final volume of 1 mL of OMV suspension. OMV isolates were inoculated into LB agar plates were incubated at 37°C for 24 hours to assess sample sterility. Isolated OMV samples were pooled by strain to obtain 5 mL OMV samples per strain. Samples were stored at 4 °C until ready to use.

### Physicochemical characterization of OMVs by Dynamic Light Scattering (DLS).

Malvern ZetaSizer Nano series ZS with 4 mW 632.8 nm laser was used to determine the average apparent hydrodynamic diameter of OMVs. Briefly, bacterial OMV samples were prepared by adding 50 μL of sample into Malvern Panalytical disposable plastic cuvettes for Zetasizer. Buffer conductivity, viscosity, and absorbance of the Qiagen Buffer XE were determined using a Mettler Toledo^®^ Refracto 30GS and used to calibrate the Malvern Panalytical ZetaSizer Nano. Diameter was measured through DLS at a 173 ° angle for 10 seconds in 13 replicates per sample.

### Physicochemical characterization of OMVs by Confocal Microscopy.

Bacterial OMVs were labeled using Invitrogen Molecular Probes^®^ Lipophilic Tracer – Vybrant^®^ DiL. Briefly, a volume of OMV suspension containing 10 μg of OMVs was mixed with 5 μL of Vybrant^®^ DiL(50 mg/mL dissolved in chloroform) per 1 mL of OMV suspension. OMVs were incubated with the lipophilic tracer for 20 minutes at 37°C. After incubation, unbound Vybrant^®^ DiI was removed by filtering through 100 KDa cutoff Amicon^®^ Ultra-0.5 Centrifugal Filters by centrifuging at 14,000 g for 15 minutes. A volume of 500 μL of LCMS-grade H_2_O was added to the filter with the OMVs and centrifuged at 14,000 g for 15 minutes to wash off excess lipophilic tracer. OMVs were washed twice by repeating this step, and OMVs were then resuspended in 1 mL of LCMS-grade H_2_O. Vybrant^®^ DiL-labeled OMVs were visualized under confocal microscopy in a Nikon^®^ Ti2 inverted microscope with Yokogawa CSU-W1 SoRa by adding 200 μL into wells in a glass clear-bottom black fluorescence 96-well plate. OMVs were observed in suspension at an excitation wavelength set to 549 nm and emission wavelength set to 565 nm. Images were analyzed using the NIS-Elements AR 6.10.01 software.

### OMVs treatment.

HeLa cells were cultured in DMEM (SIGMA) supplemented with 10% FBS (SIGMA) and Penicillin/Streptomycin (100 U:100 μg/mL) (CORNING) as previously described.

#### 4-hour exposure.

When cells reached 50% to 60% confluency, cells were exposed to 12 μg of OMVs resuspended in DMEM High Glucose supplemented with 10% FBS and 1% Pen / Strep for 4 hours. After 4 hours, OMVs were removed, cells were washed twice with homemade sterile Phosphate Buffered Saline (PBS) 1X pH 7.4 and fixed with methanol for 5 minutes. Methanol was removed and cells were washed twice with homemade sterile PBS 1X pH 7.4.

#### 72-hour exposure.

When cells reached 50% to 60% confluency, cells were exposed to 12 μg of OMVs resuspended in DMEM High Glucose supplemented with 10% FBS and 1% Pen / Strep for 4 hours. After 4 hours, OMVs were removed, cells were washed twice with homemade sterile PBS 1X pH 7.4 and fresh cell culture media was added. Cells were allowed to grow for an additional 68 hours, after which cell culture was removed, cells were washed twice with homemade sterile PBS 1X pH 7.4 and fixed with methanol for 5 minutes. Methanol was removed and cells were washed twice with homemade sterile PBS 1X pH 7.4.

### Phenotypic Analysis of HeLa cells treated with OMVs.

HeLa cells were grown on sterile round coverslips in a 24-well culture plate as previously described.

#### Giemsa Staining.

Giemsa stain was prepared by diluting Giemsa in LCMS-grade H_2_O (1:20). Fixed cells were treated with Giemsa stain:LCMS-grade H_2_O for 20 minutes. Giemsa stain was removed, and cells were washed twice with LCMS-grade H_2_O and allowed to air dry. After sample were dry, cells were covered with xylene-based mounting medium and allowed to dry overnight.

#### Microscopy Visualization of HeLa Cells.

OMV-treated HeLa cells were visualized under confocal microscopy in a Nikon^®^ Ti2 inverted microscope with Yokogawa CSU-W1 SoRa using a monochromatic filter. Cells were seen at 20X and 60X magnification at which images were taken. Images were then analyzed using the NIS-Elements AR 6.10.01 software.

### Detection of DNA damage markers in HeLa cells treated with OMVs.

HeLa cells (ATCC CCL-2) were cultured in Dulbecco’s Modified Eagle’s Medium (DMEM) High Glucose supplemented with 10% Fetal Bovine Serum (FBS) and 1% Penicillin / Streptomycin (Pen / Strep) (10,000 U/mL). Culture was done at 37 °C with 5% CO_2_. Cells were grown in T25 flasks until the fourth passage. HeLa cells were then transferred to T75 flasks for OMV exposure.

#### 4-hour exposure.

When cells reached 50% to 60% confluency, cells were exposed to 12 μg of OMVs resuspended in DMEM High Glucose supplemented with 10% FBS and 1% Pen / Strep for 4 hours. After 4 hours, OMVs were removed, cells were washed twice with homemade sterile Phosphate Buffered Saline (PBS) 1X pH 7.4 and 500 μL of Laemmli Buffer 1X with 5% of β-mercaptoethanol was added to flasks. Cells were then scraped using cell scrapers and removed from flask to place them in a 1.5 mL microtube. The scraped cells were then sonicated at a frequency of 20 kHz using a probe sonicator for 10 seconds.

#### 72-hour exposure.

When cells reached 50% to 60% confluency, cells were exposed to 12 μg of OMVs resuspended in DMEM High Glucose supplemented with 10% FBS and 1% Pen / Strep for 4 hours. After 4 hours, OMVs were removed, cells were washed twice with homemade sterile PBS 1X pH 7.4 and fresh cell culture media was added. Cells were allowed to grow for an additional 68 hours, after which cell culture was removed, cells were washed twice with homemade sterile Phosphate Buffered Saline (PBS) 1X pH 7.4 and 500 μL of Laemmli Buffer 1X with 5% of β-mercaptoethanol was added to flasks. Cells were then scraped using cell scrapers and removed from flask to place them in a 1.5 mL microtube. The scraped cells were then sonicated at a frequency of 20 kHz using a probe sonicator for 10 seconds.

### Labeling of OMVs.

Bacterial OMVs were labeled using Invitrogen Molecular Probes^®^ Lipophilic Tracer – Vybrant^®^ DiO. Briefly, a volume of OMV suspension containing 2 μg of OMVs was mixed with 5 μL of Vybrant^®^ DiO per 1 mL of OMV suspension. OMVs were incubated with the lipophilic tracer for 20 minutes at 37°C. After incubation, unbound Vybrant^®^ DiO was removed by filtering through 100 KDa cutoff Amicon^®^ Ultra-0.5 Centrifugal Filters by centrifuging at 14,000 xg for 15 minutes. A volume of 500 μL of LCMS-grade H_2_O was added to the filter with the OMVs and centrifuged at 14,000 g for 15 minutes to wash off excess unbound lipophilic tracer. OMVs were washed twice by repeating this step, and OMVs were then resuspended in 1 mL of homemade sterile PBS 1X pH 7.4. Labelling was performed under low light conditions.

### OMV internalization by Confocal Microscopy.

HeLa cells (ATCC CCL-2) were cultured in Dulbecco’s Modified Eagle’s Medium (DMEM) High Glucose supplemented with 10% Fetal Bovine Serum (FBS) and 1% Penicillin / Streptomycin (Pen / Strep) (10,000 U/mL). Culture was done at 37 °C with 5% CO_2_. Cells were grown in T25 flasks until the fourth passage. HeLa cells were counted in a BioRad TC20^™^ Automated Cell Counter using Trypan Blue. A total of 1.0 × 10^4^ cells were transferred to individual wells in a 24-well plate.

#### Exposure to Bacterial Vybrant^®^ DiO-labeled OMVs (4 hours).

When cells reached 50% to 60% confluency, they were exposed to 2 μg of Vybrant^®^ DiO-labeled OMVs resuspended in DMEM High Glucose supplemented with 10% FBS and 1% Pen / Strep for 4 hours. After 4 hours, OMVs were removed, cells were washed twice with homemade sterile Phosphate Buffered Saline (PBS) 1X pH 7.4 and fixed with 4% paraformaldehyde for 20 minutes. The paraformaldehyde was removed, and cells were washed twice with homemade sterile PBS 1X pH 7.4.

#### Exposure to Bacterial Vybrant^®^ DiO-labeled OMVs (72 hours).

When cells reached 50% to 60% confluency, cells were exposed to 2 μg of Vybrant^®^ DiO-labeled OMVs resuspended in DMEM High Glucose supplemented with 10% FBS and 1% Pen / Strep for 4 hours. After 4 hours, OMVs were removed, cells were washed twice with homemade sterile PBS 1X pH 7.4, and fresh cell culture media was added. Cells were allowed to grow for an additional 68 hours, after which cell culture was removed, cells were washed twice with homemade sterile PBS 1X pH 7.4 and fixed with 4% paraformaldehyde for 20 minutes. The paraformaldehyde was removed, and cells were washed twice with homemade sterile PBS 1X pH 7.4.

#### Cellular labelling.

Cells were permeabilized with 0.1% Triton X-100 in homemade sterile PBS 1X pH 7.4 for 3 minutes. Afterwards, cells were washed twice with homemade sterile PBS 1X pH 7.4 and 50 μL of Phalloidin-TRITC (2 μg/μL) were added to each well. Cells were treated for 20 minutes before washing twice with homemade sterile PBS 1X pH 7.4. Cells treated with Vybrant^®^ DiO-labeled OMVs and labelled with Phalloidin-TRITC were treated with 50 μL of DAPI (1 mg/mL) for 20 minutes. Afterwards, cells were washed twice with homemade sterile PBS 1X pH 7.4. After nuclei labelling and washing, cells were covered in mounting medium and allowed to dry overnight in protected from light. Labelling was performed under low light conditions.

Fluorescently labelled cells and OMVs were visualized under confocal microscopy in a Nikon^®^ Ti2 inverted microscope with Yokogawa CSU-W1 SoRa. Excitation and emission wavelengths used for each of the fluorescent markers are listed in **Table 2**.

### Statistical Analysis.

Values were reported as means ± standard error means of biological replicates. *p*-values were calculated using one-way or two-way (according to each experiment) analyses of variance (ANOVA) with the “Tukey’s Multiple Comparison” test for multiple comparisons. All statistical analyses were conducted using Prism 10 software (GraphPad, La Jolla, CA, United States).

## Supplementary Material

Additional Information

Supporting information

This article contains all the supporting information.

## Figures and Tables

**Table 1:** Average concentrations and diameters of pooled OMV samples.

Sample	Concentration (μg/mL)	Std. Dev. (μg/mL)	Diameter (nm)	Std. Dev. (nm)
NC101	83.0	3.0	135.3	9.7
NC101Δ*clbP*	76.2	0.3	164.3	31.4
DH10B	99.6	2.6	122.4	0.0

## Data Availability

All data discussed here are presented in the manuscript.
